# Studying the Expansion Kinetics of a Single Bead–Spring Chain Under Theta Conditions in 3D and 2D Spaces

**DOI:** 10.3390/polym18182235

**Published:** 2026-09-13

**Authors:** Pai-Yi Hsiao

**Affiliations:** 1 Department of Engineering and System Science, National Tsing Hua University, Hsinchu 300044, Taiwan; pyhsiao@mx.nthu.edu.tw; Tel.: +886-3-516-2247; 2 Institute of Nuclear Engineering and Science, National Tsing Hua University, Hsinchu 300044, Taiwan

**Keywords:** polymer expansion, theta solvent, scaling theory, molecular simulations

## Abstract

Expansion of a polymer chain released from a confined state under theta conditions is investigated by means of Langevin dynamics simulations in both three- and two-dimensional geometries. Using a bead–spring chain representation, the θ-temperature is first identified through a scaling analysis of the chain size that exhibits the expected power-law behavior. Extensive simulations then carried out at this temperature reveal that the time evolution of the average chain size during expansion displays sigmoidal curves on log–log plots, indicating a two-stage process: an initial rapid power-law expansion from the confining size, followed by a slower exponential relaxation toward the final equilibrium size. By examining the regularity of these curves, we determine the scaling laws that govern the characteristic times and expansion exponents in each stage. The 2D expansion is found to proceed with slower dynamics than the 3D case, owing to the restriction of the available chain motion on a plane. Plotting the expansion rate against chain size provides a direct test of the kinetic equations, producing master evolutionary trajectories for both stages. Integrating these kinetic equations yields the scaling forms of the free energy for each stage, from which several relationships between the scaling exponents are obtained. This study demonstrates that incorporating local steric constraints and prohibiting segment crossing is essential for accurately describing chain expansion kinetics at the theta point, thereby exposing the limitations of idealized freely jointed chain models.

## 1. Introduction

In polymer science, the “theta condition” is a special state where a polymer chain behaves as if it were ideal [1,2]. At this specific solvent condition or temperature, the polymer–polymer interactions exactly cancel the polymer–solvent interactions, producing a zero second virial coefficient. The theta condition thus serves as a crucial reference point for benchmarking theoretical models and deepening our understanding of fundamental polymer properties [3,4,5]. For instance, the intrinsic chain size can be measured at the theta temperature, effectively eliminating solvent-dependent perturbations. Characterization techniques such as intrinsic viscosity, osmotic pressure, and light-scattering measurements yield consistent results across different theta systems, providing a reliable standard for comparison [6,7,8,9].

While a polymer at the theta condition shows static statistics and size-scaling behavior similar to an ideal chain, subtle differences still exist between the two systems [1,10,11]. A real chain always experiences nearby interactions that prohibit monomer overlap and segment crossing. By contrast, an ideal chain is assumed to be interaction-free and behaves as a “phantom chain”—an abstract, purely mathematical model that permits overlap and crossing. Therefore, it remains unclear to what extent the dynamics of a polymer at theta conditions can be captured by an ideal-chain description. In fact, in two-dimensional space, self-avoidance between monomers becomes a non-negligible effect at the θ-point, compared with the three-dimensional space. Consequently, the chain-size scaling deviates from the 3D form of $R∼N^{1/2}$ to the 2D form of $R∼N^{4/7}$ [12,13,14,15]. In this study, we examine the expansion kinetics of a flexible polymer released from spherical confinement under theta conditions. A comprehensive understanding of this phenomenon will not only provide a theoretical framework for polymer physics, but also shed light on biological processes such as the release of genomes from small particles or viral capsids in microbiology and virology [16,17,18]. Specifically, DNA molecules or therapeutic drugs are frequently packaged into nanoscale carriers for delivery. Knowledge of the release mechanisms is crucial for subsequent biomedical applications and for the development of advanced nanotechnologies [19,20,21,22].

The transition of a polymer between a collapsed (globular) state and an extended (coiled) state has long been a central topic in polymer science [2,23,24,25,26]. By improving the solvent quality, a collapsed chain can be driven into an extended conformation. Conversely, a deteriorated solvent can collapse an extended chain. These globule-to-coil (GTC) and coil-to-globule (CTG) transitions have attracted considerable attention. In the 1990s, Wu and collaborators cyclically varied the temperature and studied single-chain transitions [27,28]. They observed hysteresis in the transitions and identified a range of intermediate conformations including coil, crumpled coil, molten globule, and globule. A two-stage GTC theory was subsequently developed by Pitard and Orland [29], and stated $R^{2}\left(t\right)≃R^{2}\left(0\right)\left(1+{\left(\frac{t}{\tau_{c}}\right)}^{3/4}\right)$ when $t\ll \tau_{1}$ and $R\left(t\right)∼N^{3/5}\left(1-\exp\left(-\frac{t}{\tau_{1}}\right)\right)$ when $t\gg \tau_{1}$ where the two characteristic time scales scale as $\tau_{c}∼N^{4/3}$ and $\tau_{1}∼N^{11/5}$. The role of chain entanglement during the GTC transition has also been investigated [30,31]. In a dry globule, reptation becomes the dominant mechanism for disentangling the chain, and the swelling time scales as $N^{3}$. If the globule is hydrated, the process proceeds through an arrested intermediate and the transition time scale reduces to $N^{2}$. Without entanglement, the swelling time follows power-law dynamics: $t^{1/5}$ for a wet globule and $t^{2/5}$ for a dry globule. Later, Sakaue and Yoshinaga approached the GTC problem from a thermodynamic perspective [32]. They predicted that the chain size evolves as $R\left(t\right)≃R_{0}{\left(1+\frac{t}{\tau_{\mathrm{ini}}}\right)}^{\alpha }$ with $\alpha =\frac{3\nu -1}{9\nu }$ and $\tau_{\mathrm{ini}}≃\tau_{0}{\left(\frac{R_{0}}{aN^{1/3}}\right)}^{1/\alpha }$ where ν is the Flory exponent, *a* is the monomer size, and $\tau_{0}$ is a microscopic time scale.

Recently, we addressed the problem in a different way: a polymer chain expands athermally after being released from a tightly confining state [33,34]. Unlike a GTC transition, the solvent quality remains fixed here, so the chain’s expansion is a non-equilibrium thermodynamic process. The evolution is predicted to follow the two distinct regimes: (1) a power-law expansion $R\left(t\right)=R_{I}{\left(1+\frac{t}{\tau_{1}}\right)}^{\alpha_{1}}$ for $t≲\tau_{1}$, and (2) an exponential relaxation $R\left(t\right)=R_{F}{\left(1-\exp\left(-\frac{t}{\tau_{2}}\right)\right)}^{\alpha_{2}}$ for $t≳\tau_{2}$. Here, $R_{I}$ and $R_{F}$ denote the initial and final equilibrium sizes, while $\alpha_{1}$ and $\alpha_{2}$ are the two scaling exponents and $\tau_{1}$ and $\tau_{2}$ the corresponding characteristic times. Given the spatial dimension *d*, the initial confining volume fraction $\phi_{0}$, and the Flory exponent $\nu_{b}$ of a blob, we found $\alpha_{1}=\frac{d\nu_{b}-1}{2(d\nu_{b}-1)+d}$, $\alpha_{2}=\frac{1}{d+2}$, $\tau_{1}∼N^{\frac{2}{d}+\chi_{1}}\phi_{0}^{-\frac{1}{d\alpha_{1}}}$, and $\tau_{2}∼N^{2+\chi_{2}}$. The additional exponents $\chi_{1}$ and $\chi_{2}$ account for the extra scaling of the effective friction coefficient associated with the expansion velocity. These relations were derived from a theory that balances the free energy loss against the energy dissipation, and have been verified by extensive molecular simulations.

We extended the study to the expansion of an ideal chain released from a spherical confinement [35]. Within the freely jointed chain framework, the chain again exhibits a two-stage expansion, but the exponents are universal: $\alpha_{1}=\frac{1}{3}$ and $\alpha_{2}=\frac{1}{4}$ in both two and three dimensions. The corresponding characteristic times turn out to be $\tau_{1}∼{\left(\frac{R_{I}}{\sigma }\right)}^{3}$ and $\tau_{2}∼N^{2}$, which are independent of dimensionality. However, as noted earlier, the expansion kinetics of a real polymer at the theta condition can differ from those of an ideal chain. Therefore, it is important to investigate theta behaviors using a chain model that incorporates appropriate local interactions to prevent monomer overlap and self-crossing.

Several approaches exist for simulating polymer chains under different solvent conditions. The most detailed method explicitly includes solvent molecules in the simulation. In 1989, Smit et al. [36] investigated how the solvent quality, tuned by varying the polymer-solvent interaction, influences the properties of short polymer chains. A similar model has recently been used to study conformational changes of multiple long chains and phase separation in polymer solutions [37]. The behavior of several polyelectrolyte chains at overlap concentrations has also been examined using the TIP4P water model [38]. By varying the Flory–Huggins interaction parameter, the theta point and the CTG transitions of flexible homopolymers were found to occur at higher temperatures in explicit-solvent models than in implicit-solvent ones, as demonstrated by Monte Carlo simulations [39].

The computational efficiency can be significantly enhanced by dissipative particle dynamics (DPD) simulations, because a DPD solvent bead represents a cluster of several solvent molecules, thereby largely reducing the modeling complexity [40,41,42]. Kong et al. [43] demonstrated that the CTG transition of polymers can be investigated within DPD by systematically tuning the repulsion parameter to vary the solvent quality. The influence of solvent quality on the dynamics of polymer solutions has also been explored [44,45]. DPD studies have shown that polymer translocation through a nanopore is slowed in a good solvent compared with a poor solvent [46]. Near the theta point, polymer knots exhibit pronounced breathing motions, which accelerate the unknotting dynamics [47].

To reduce computational cost even further, polymer solutions can be modeled with implicit solvent approaches. The dimensions and conformational properties of long chains and star polymers have been examined by Monte Carlo simulations in which the effective monomer–monomer interaction is tuned to emulate different solvent qualities [48,49,50]. The structure of polymer brushes and their tribological properties have been studied under various solvent conditions by adjusting the depth of the Lennard–Jones potential in Langevin dynamics simulations [51,52]. The influence of solvent quality on the coil-stretch transition of polymers in flow has been investigated through Brownian dynamics simulations [53]. Owing to topological constraints, the θ-temperature for a ring polymer is found to be smaller than that for a linear chain [54]. The kinetics of the end-to-end contact of a chain have been investigated: while large chain stiffness generally destabilizes the end-to-end contact state, poor solvents stabilize it [55]. Molecular dynamics simulations showed that the radial distributions and diffusion behavior of star polymers can be described by universal functions in solvents of various quality [56]. Within the implicit solvent framework, the properties of poly[n]catenane systems were studied by varying the interaction cutoff $r_{\;c}$, which effectively alters the monomer excluded volume; a depression of the θ-temperature compared with both ring and linear polymer counterparts was observed [57,58,59].

So far, studies of the expansion kinetics of a confined chain released under θ-conditions are still scarce and the phenomenon demands detailed investigation. In this work, we tackle the problem by performing extensive molecular simulations using a bead–spring chain model in which the monomers are represented by non-penetrating beads—a crucial detail for realistic modeling (see Section 2 for the model and setup). Using an implicit-solvent approach, the θ-temperature of the chain system is identified by varying the temperature via a scaling analysis of the chain size (see Section 3.1). The chain expansion is then studied at the θ-temperature in both three-dimensional and two-dimensional spaces (Section 3.2). The sigmoidal variation in the chain size in a log–log plot demonstrates that the expansion proceeds in two distinct stages, each characterized by its own expansion exponent and time. Analysis of the expansion speed (Section 3.3), combined with the chain-size evolution, further reveals a universal variation pattern. With the aid of Onsager’s variational theory, we extract the scaling form of the free energy during the two expansion stages (see Section 3.4), which differs markedly from that of an ideal chain predicted by the freely jointed chain model. We discuss these findings and present our conclusions in Section 4.

## 2. Model and Setup

Molecular dynamics simulations are performed to investigate the expansion of a spherically confined chain upon sudden release in three-dimensional and two-dimensional spaces, using a bead–spring chain (BSC) model. A chain consists of N+1 monomer beads, linearly connected by *N* bonds. A bond is modeled by a spring potential, defined by $U_{\mathrm{sp}}\left(b_{i}\right)=\frac{1}{2}k_{\mathrm{sp}}{\left(b_{i}-b_{0}\right)}^{2}$ where $k_{\mathrm{sp}}$ is the spring constant, $b_{0}$ is the equilibrium length, and $b_{i}=\left|r_{i}-r_{i-1}\right|$ is the length of the *i*th bond between the adjacent monomers i−1 and *i*. Any pair of the non-bonded monomers, *i* and *j*, interact with each other via the Lennard–Jones potential

(1) $$U_{\mathrm{LJ}}\left(r_{ij}\right)=\left\{\begin{array}{cc} 4\varepsilon \left[{\left(\frac{\sigma }{r_{ij}}\right)}^{12}-{\left(\frac{\sigma }{r_{ij}}\right)}^{6}\right] & \mathrm{if}\;r_{ij}\leq 2.5\sigma \\ 0 & \mathrm{if}\;r_{ij}>2.5\sigma \end{array}\right.$$

where $r_{ij}=\left|r_{i}-r_{j}\right|$ is the distance between the monomer pair, and ε and σ define, respectively, the interaction strength and length scale. This interaction model refers to the approach described in Ref. [60]. The choice of a Lennard--Jones cutoff of 2.5σ strikes a balance between computational efficiency and physical accuracy, and has been employed in many foundational molecular dynamics studies.

The total potential energy of the system is thus $U_{\mathrm{tot}}\left({\left\{r_{i}\right\}}_{i=0}^{N}\right)=\sum_{i=1}^{N}U_{\mathrm{sp}}\left(b_{i}\right)+\sum_{i=0}^{N}\sum_{j=i+1}^{N}U_{\mathrm{LJ}}\left(r_{ij}\right)$. A Langevin thermostat is applied to control the temperature at the desired value *T*, and the dynamics of the chain are determined by the equations of motion

(2) $$m\frac{d^{2}r_{i}}{dt^{2}}=-\eta_{0}\frac{dr_{i}}{dt}-\frac{\partial U_{\mathrm{tot}}}{\partial r_{i}}+\xi_{i}$$

for i=0,⋯,N, where *m* is the mass of a monomer and $\eta_{0}$ is the friction coefficient. The term $-\eta_{0}\frac{dr_{i}}{dt}$ is the dragging force acting on the monomer *i* when it passes through the solvent, and $\xi_{i}$ is a stochastic force simulating the random collision with the implicit solvent molecules. $\xi_{i}$ has zero mean and its variance satisfies the fluctuation-dissipation theorem, $\langle \xi_{i}\left(t\right)\cdot \xi_{j}\left(t^{\prime }\right)\rangle =2dk_{B}T\eta_{0}\delta_{ij}\delta \left(t-t^{\prime }\right)$, in the *d*-dimensional space.

We set the parameters $k_{\mathrm{sp}}$ and $b_{0}$ for the spring potential to be $12000\;\varepsilon /\sigma^{2}$ and 1.0σ, respectively. The friction coefficient $\eta_{0}$ is set to $20.0\;\sqrt{m\varepsilon }/\sigma$ to simulate the aqueous condition. In this study, we investigate chain expansion under theta condition. Therefore, the θ-temperature, $T_{\theta }$, has to be determined first. To achieve the goal, the scaling properties of chain in a bulk solution are investigated by varying the temperature. The value of *T* is varied from 1.0 to $5.0\;\varepsilon /k_{B}$ for the 3D study, whereas it is varied from 0.5 to $2.0\;\varepsilon /k_{B}$ for the 2D case. The simulations are conducted using the LAMMPS package (http://lammps.sandia.gov/, accessed on 2 August 2026) [61], employing the Verlet algorithm with an integration time step of $\Delta t=0.005\;\sigma \sqrt{m/\varepsilon }$. In the literature, θ-behavior can be examined in different ways [49,50,51,52,53,54,55,56,57,58,59]. One common strategy is to vary the interaction strength or the cutoff while keeping the temperature fixed, thereby mimicking changes in solvent quality through mixing or titration. In the present work, the solvent quality is varied by changing the temperature, not by altering the interaction potential itself. Consequently, the functional form of the interaction (including the 2.5σ cutoff) is unchanged during the study.

Once $T_{\theta }$ is determined, we confine a chain into a cavity and equilibrate it at the θ-temperature. The cavity is a sphere in the 3D space, while it is a disk in the 2D space. The wall of the cavity reflects the monomer beads at the bead radius $r_{b}=0.5\;\sigma$. The confining condition is defined by the volume fraction given by $\phi_{d}=\left(N+1\right){\left(\frac{\sigma }{D}\right)}^{d}$ where *D* is the diameter of cavity. To begin with the expansion, the reflection interaction of the cavity wall is switched off suddenly. The confined chain undergoes a free expansion and eventually reaches its natural size in the bulk solution. To investigate the kinetics of expansion systematically, the chain length *N* is varied from 16 to 512, as the power of 2. Various confining conditions are studied: $\phi_{3}=0.4$, 0.2, 0.1 for the 3D expansion and $\phi_{2}=0.6$, 0.3, 0.15 for the 2D expansion.

The physical quantities given above have been described using the metric system of simulation, which are ε for the energy, σ for the length, and *m* for the mass, respectively. In order to reduce the length of notation, the unit of any physical quantity in the following text will not be displayed any longer. Without special mention, the non-displayed unit is directly derived from the three basic simulation units.

## 3. Results and Discussion

### 3.1. Determination of $T_{\theta }$

Under the theta condition, a chain behaves similarly to an ideal chain. A straightforward method to determine the θ-temperature, $T_{\theta }$, is to examine how the chain size scales with the chain length *N*, looking for the $N^{1/2}$ dependence. However, this ideal-chain scaling applies only in the 3D space. In 2D space, self-avoidance between monomers alter the scaling at the theta point to $N^{4/7}$, as shown in Refs. [12,13,14,15].

We determine the chain size in solution by evaluating the equilibrium average squared radius of gyration with

(3) $$\langle R_{g}^{2}\rangle =\frac{1}{N+1}\langle \sum_{i=0}^{N}{\left(r_{i}\left(t\right)-r_{\mathrm{cm}}\left(t\right)\right)}^{2}\rangle ,$$

where $r_{i}\left(t\right)$ is the position of the monomer *i* at time *t*, and $r_{\mathrm{cm}}\left(t\right)$ is the instantaneous center of mass of the chain. The brackets denote an average taken over the simulation time and over an ensemble of 1000 independent runs at the temperature *T*. According to the theory, the ratio $\langle R_{g}^{2}\rangle /N^{2\nu_{\theta }}$ should be independent of the chain length *N* at the theta point with the critical exponent $\nu_{\theta }=1/2$ in three dimensions and $\nu_{\theta }=4/7$ in two dimensions. Consequently, when this quantity is plotted against *N*, it should flatten out at $T=T_{\theta }$.

**Figure 1:**
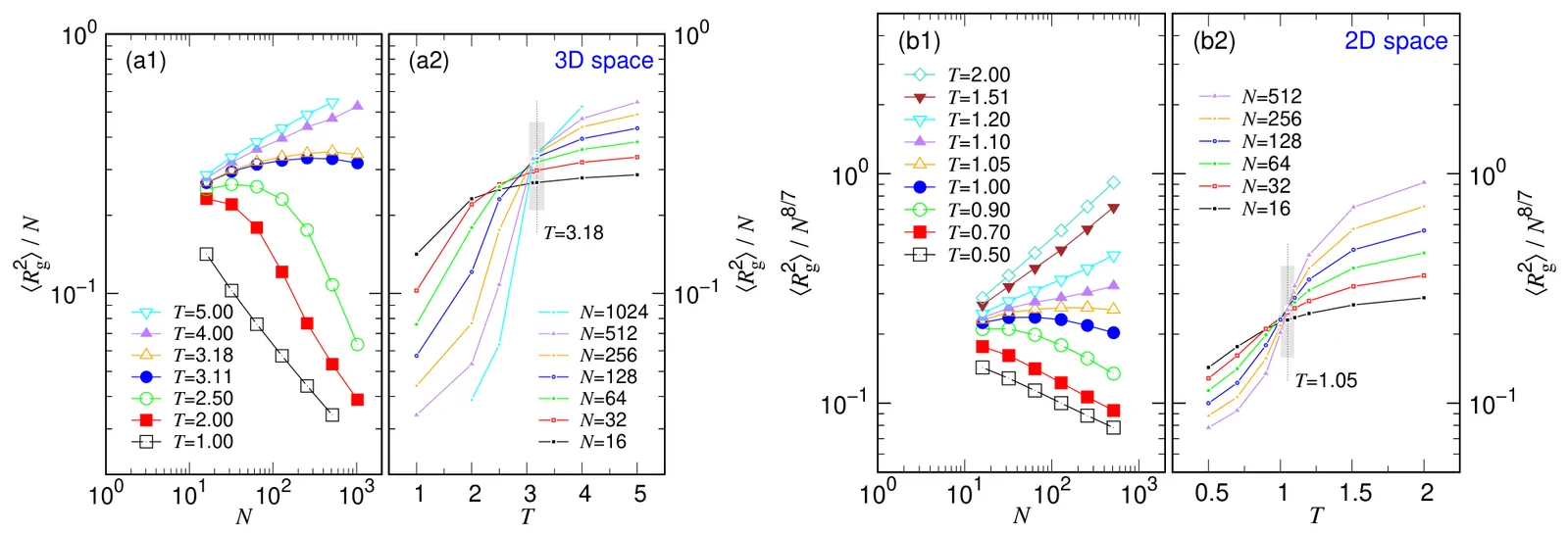
Plots of $\langle R_{g}^{2}\rangle /N^{2\nu_{\theta }}$ versus *N* with (**a1**) $\nu_{\theta }=1/2$ in 3D space and (**b1**) $\nu_{\theta }=4/7$ in 2D space, at various temperatures *T*, as indicated in the legends. Additionally, plots of $\langle R_{g}^{2}\rangle /N^{2\nu_{\theta }}$ as a function of *T* for different *N*, with values read from the legends, are shown in (**a2**) for the 3D system and (**b2**) for the 2D system. The dashed lines mark the temperatures 3.18 in 3D and 1.05 in 2D. Grey strips represent the regimes with a marginal error of 4.4% associated to the temperature determination.

By varying the temperature, we found that the quantity $\langle R_{g}^{2}\rangle /N^{2\nu_{\theta }}$ becomes approximately constant at T=3.18 in 3D and at T=1.05 in 2D, as shown in Panels (a1) and (b1) of Figure 1. Because the scaling relation is valid only in the long-chain limit, the leveled-off, constant behavior is visible for N>100

The plot in Panel (a2) exhibits that the curves of $\langle R_{g}^{2}\rangle /N^{2\nu_{\theta }}$ versus *T* intersect around T=3.18 when N≥128, suggesting again that $T_{\theta }≅3.18$. The finding is consistent with the results of a previous study that used a similar model [60]. Similarly, in the 2D space, the $\langle R_{g}^{2}\rangle /N^{2\nu_{\theta }}$ curves for different *N* tend to intersect between each other at T≅1.05, as shown in Panel (b2). Therefore, we chose $T_{\theta }≅1.05$ for the 2D space.

We remark that in the plots, the curves for N=16, 32, and 64 are also presented to illustrate how the behavior evolves for shorter chains. The pronounced finite-size effects at these lengths may give an impression that the intersections are not converging well. However, the theta regime is known to span a temperature window given by $T_{\theta }\left(1-\frac{1}{\sqrt{N}}\right)<T<T_{\theta }\left(1+\frac{1}{\sqrt{N}}\right)$ [11,59]. For N=512, the width $\frac{1}{\sqrt{N}}$ corresponds to a marginal error of about 4.4% in the θ-temperature. Panels (a2) and (b2) show that the intersections between the curves for long chains converge within these windows (indicated in grey strip in the two figures). Therefore, the two chosen temperatures lie within the θ-behavior window and can be used to study polymer expansion later.

**Figure 2:**
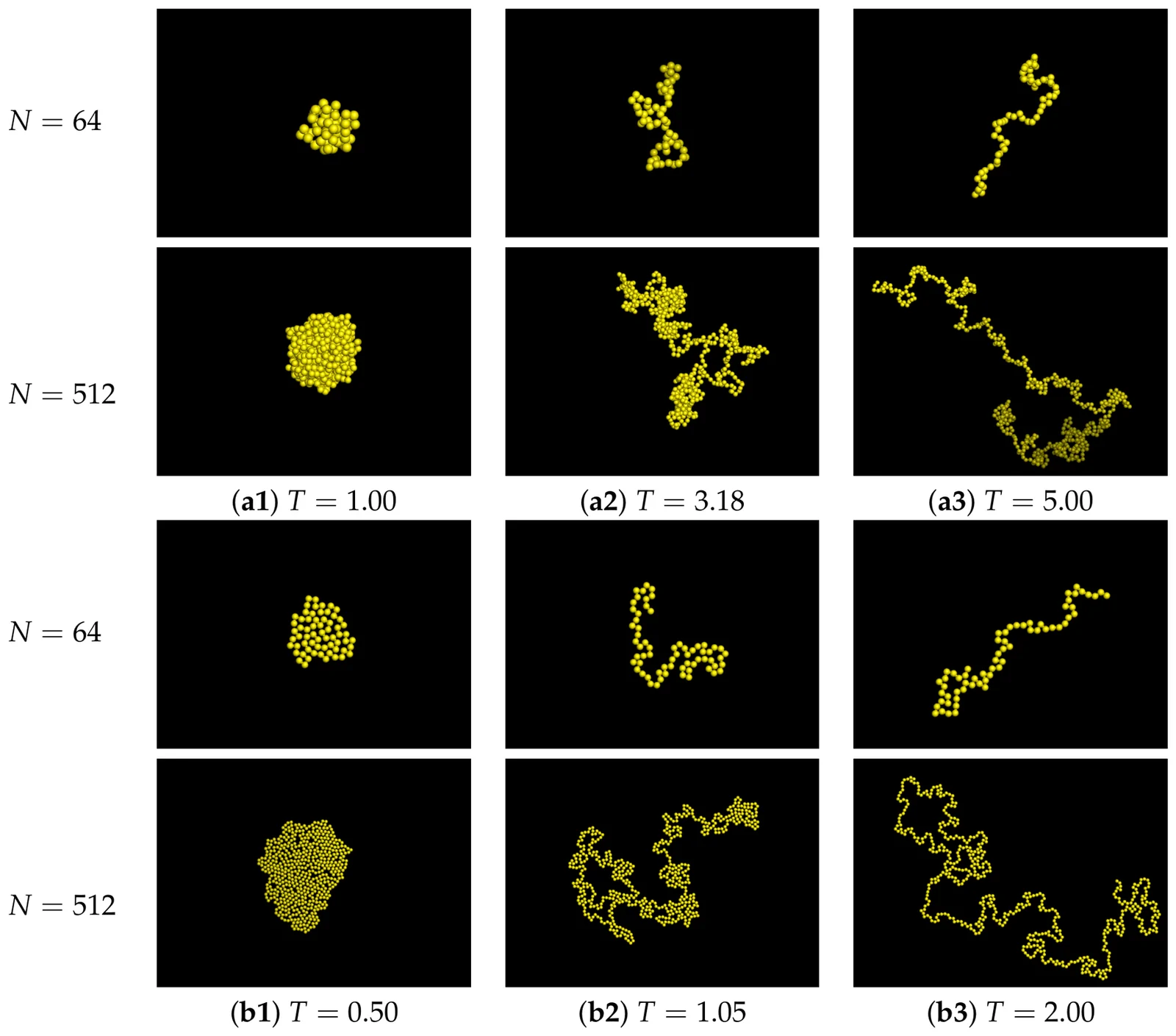
Snapshots of a chain with length N=64 and 512 in equilibrium within bulk solutions are shown with (**a1**–**a3**) displaying the 3D configurations and (**b1**–**b3**) illustrating the 2D configurations. The temperature corresponding to each snapshot is indicated below it.

Snapshots of a chain with N=64 and 512 in equilibrium at temperatures below, equal to, and above $T_{\theta }$ are shown in Figure 2.

We observe that at $T\ll T_{\theta }$, such as T=1.0 in the 3D space and T=0.5 in the 2D space, both the long and the short chains collapse into a compact, small-volume conformation. Conversely, for $T\gg T_{\theta }$ like T=5.0 in 3D and T=2.0 in 2D, the chains adopt an extended, swollen state. Near the transition, specifically, T=3.18 in 3D and T=1.05 in 2D, the long and short chains both display a coil-like structure with locally aggregated monomers, attributable to the attractive branch of the Lennard–Jones potential acting between neighboring monomers.

**Figure 3:**
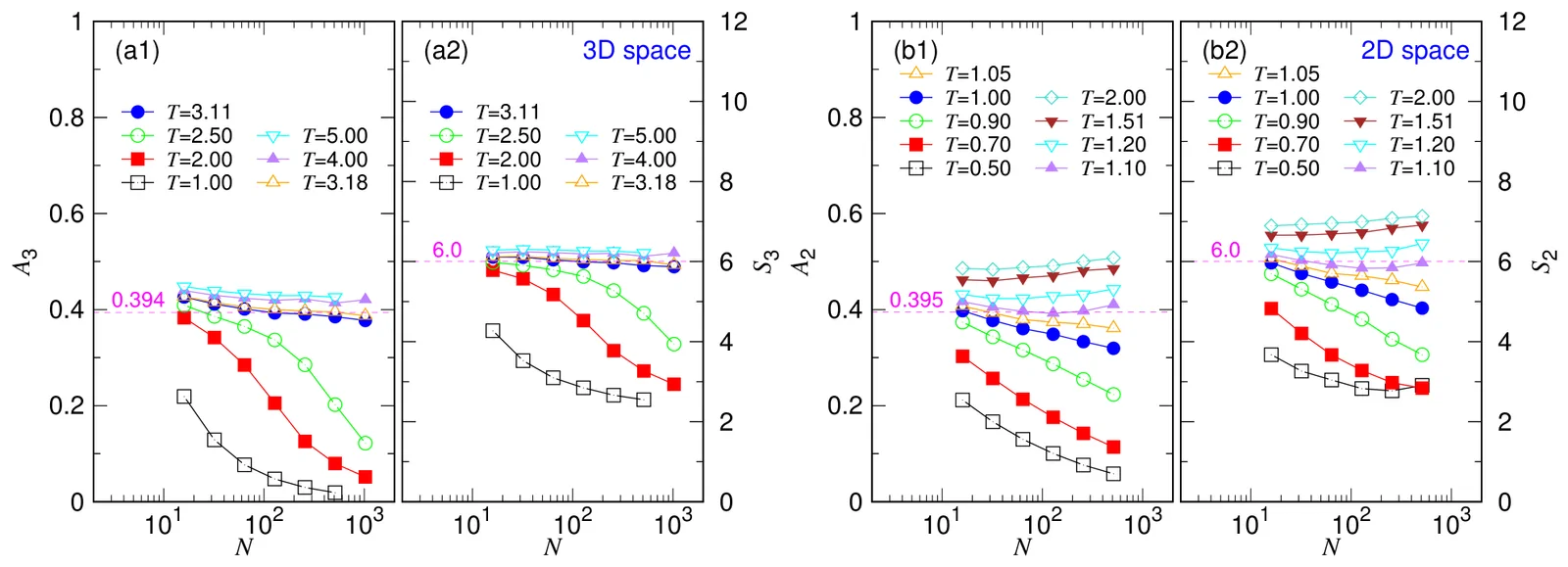
Asphericity $A_{d}$ and shape factor $S_{d}$ as functions of *N* in *d*-dimensional space: (**a1**,**a2**) for d=3, and (**b1**,**b2**) for d=2. The temperature *T* values can be read from the legends.

The chain conformation at different temperatures can be studied by calculating the asphericity $A_{d}$ and the shape factor $S_{d}$ in *d*-dimensional space [62], defined as follows:

(4) $$\begin{array}{ccc} A_{d} & = & \langle \frac{\sigma_{\lambda }^{2}}{d{\bar{\lambda }}^{2}}\rangle , \end{array}$$

(5) $$\begin{array}{ccc} S_{d} & = & \frac{\langle R_{e}^{2}\rangle }{\langle R_{g}^{2}\rangle }, \end{array}$$

where $\sigma_{\lambda }^{2}=\frac{1}{d-1}\sum_{\alpha =1}^{d}{\left(\lambda_{\alpha }-\bar{\lambda }\right)}^{2}$ is the sample variance of the eigenvalues $\lambda_{\alpha }$ (for α=1,⋯,d) of the gyration tensor,

(6) $$\left[Q_{\alpha \beta }\right]=\left[\frac{1}{N+1}\sum_{i=0}^{N}\left(r_{i,\alpha }-r_{\mathrm{cm},\alpha }\right)\left(r_{i,\beta }-r_{\mathrm{cm},\beta }\right)\right],$$

and $\bar{\lambda }=\frac{1}{d}\sum_{\alpha =1}^{d}\lambda_{\alpha }$ is the mean of the eigenvalues. $\langle R_{e}^{2}\rangle$ represents the average of the square of the end-to-end distance of the chain. The results are presented in Figure 3 as a function of *N*.

In Panel (a1), we observe that the 3D asphericity $A_{3}$ remains approximately constant, very close to the FJC value of 0.394 [35,62,63] at the obtained $T_{\theta }=3.18$. The calculation also shows that the shape factor $S_{3}$ in Panel (a2) attains the ideal-chain value of 6.0 at this temperature. In the region of lower temperature $T<T_{\theta }$, $A_{3}$ decreases, indicating a transition towards a globular structure, and the chain exhibits a more spherical conformation as *N* increases. Conversely, at higher temperature $T>T_{\theta }$, the results suggest the formation of a more extended conformation than that of an ideal-chain coil.

In the 2D case, Panels (b1) and (b2) display similar trends of variation. However, at the obtained θ-temperature $T_{\theta }=1.05$, both $A_{2}$ and $S_{2}$ are smaller than the ideal-chain values of 0.395 and 6.0, respectively [35,62]. A combination of local excluded-volume effects and Lennard--Jones attractive interactions leads to these deviations. The discrepancy is more pronounced in two dimensions because chain segments are confined to a plane and cannot cross each other due to self-avoidance, unlike in an ideal chain. Consequently, the accessible ensemble of chain configurations is more restricted, resulting in a less extended shape. A higher temperature is therefore required for the BSC model to produce $A_{2}$ and $S_{2}$ values that approach those of an ideal chain, which explains the observed deviation. Because the chain size scales as $N^{4/7}$ at the θ point in 2D space, the BSC model is not expected to behave like the FJC model.

In the following subsections, we will focus our study on the expansion-upon-release phenomena of confined single chains at the θ-temperature, specifically, T=3.18 in the 3D space and T=1.05 in the 2D space.

### 3.2. Variation in Chain Size During Expansion at $T_{\theta }$

**Figure 4:**
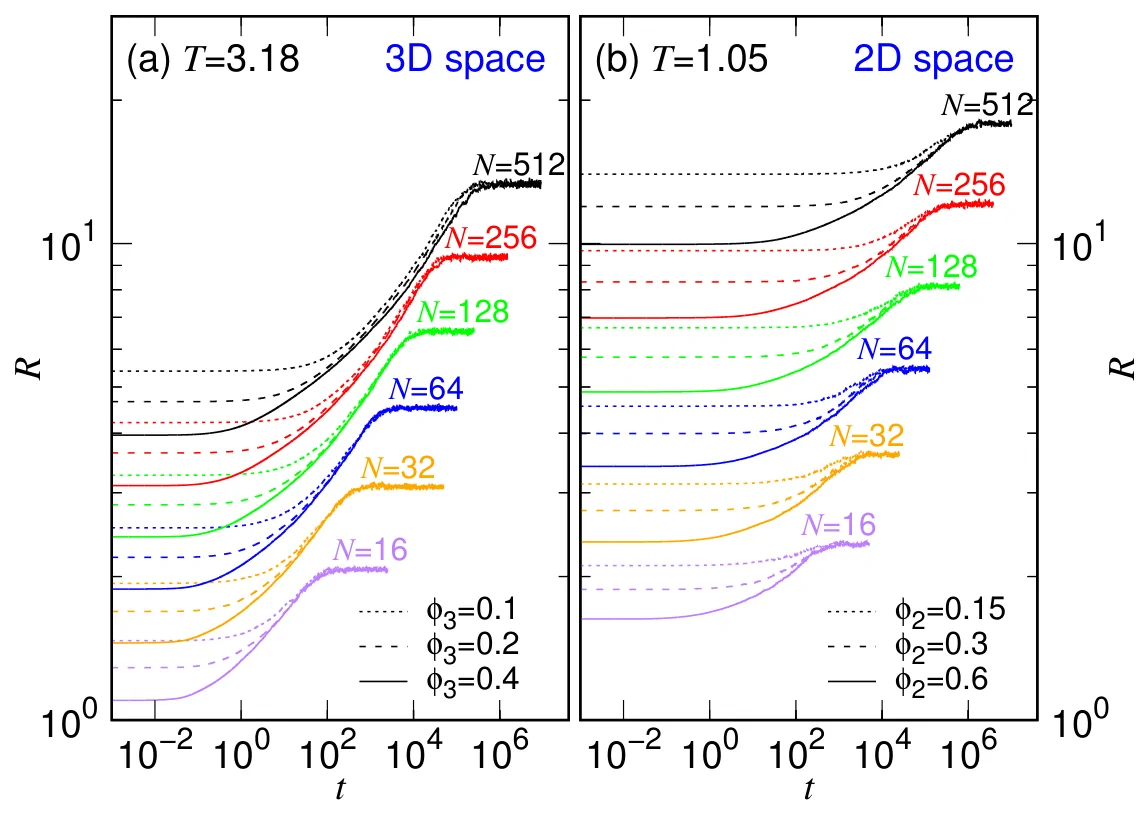
Evolution of chain size R(t) in the study of (**a**) 3D and (**b**) 2D expansions. The chain length *N* is indicated near the corresponding curves. For each chain length, three initial confinement conditions $\phi_{d}$ are examined, represented by solid, dashed, and dotted lines as shown in the legends. The temperature is fixed at T=3.18 for the 3D simulations and T=1.05 for the 2D simulations.

We first investigate the evolution of chain size, denoted by $R\left(t\right)={\langle R_{g}^{2}\left(t\right)\rangle }^{1/2}$, during an expansion process at the θ-temperature. The mean square of the radius of gyration of chain, $\langle R_{g}^{2}\left(t\right)\rangle$, is calculated by Equation (3) at every time stamp, where the average is obtained from 1000 independent runs for each studied case. The results are presented in Figure 4, for the 3D expansion in Panel (a) and 2D expansion in Panel (b) for various confining conditions and chain lengths.

The size variation displays a sigmoidal curve on log–log plots, indicating a two-stage process that changes the chain size from its initial value $R_{I}$ to the final value $R_{F}$. Different bending positions and curvature states reveal that each stage has its own characteristic time and scaling exponent.

For a given chain length *N*, stronger confinement, i.e., a larger value of $\phi_{d}$, produces a smaller initial size $R_{I}$. The R(t) curves for different confinement strengths evolve over time and eventually converge to a final value $R_{F}$ equal to the natural size of the chain in bulk solution, which is obviously independent of the initial confining condition. The plots are displayed on log–log scales because the observed time scales span several orders of magnitude. Moreover, the chain length *N* and the confinement parameter $\phi_{d}$ are varied in powers of two, respectively, to systematically investigate the scaling behavior.

**Figure 5:**
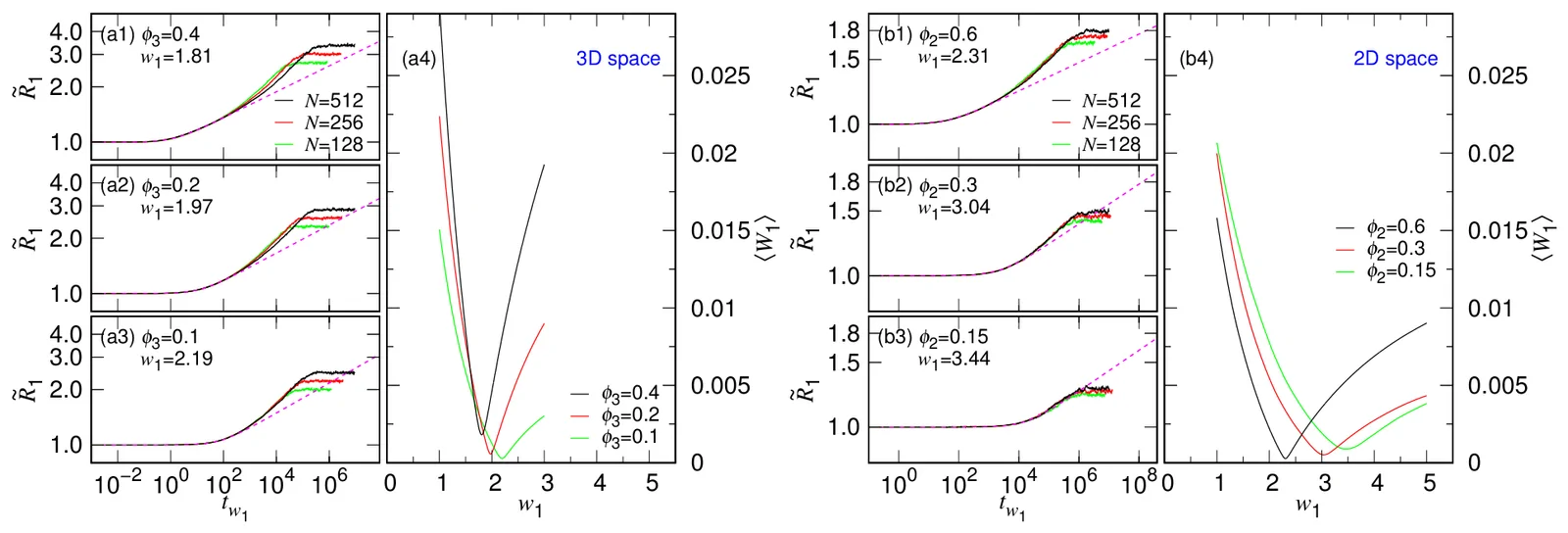
Collapse of the ${\tilde{R}}_{1}$ vs. $t_{w_{1}}$ curves in the first stage for (**a1**–**a3**) the 3D expansion cases and (**b1**–**b3**) the 2D expansion cases. The confining volume fraction $\phi_{d}$ and the $w_{1}$ value used to achieve the collapse are indicated in each panel. The chain length *N* is shown in the legends. The magenta dashed line represents a fit obtained via the equation ${\tilde{R}}_{1}\left(t\right)={\left(1+\frac{t}{\tau_{1}}\right)}^{\alpha_{1}}$. The variations in $\langle W_{1}\rangle$ vs. $w_{1}$ in the two spatial dimensions for the different $\phi_{d}$ are plotted in Panels (**a4**,**b4**).

Despite the differences in magnitude, the sets of curves for different *N* show a remarkable similarity, especially around the bending regions in the first and second stages. To investigate this similarity, we rescaled the chain size by the initial size, defining ${\tilde{R}}_{1}=R/R_{I}$, and shifted the ${\tilde{R}}_{1}$ curves horizontally along the logarithmic time axis so that they collapse onto a single target curve for the first stage. The shift was performed by multiplying time by a factor $w_{1}^{\;g_{N_{0}}-g_{N}}$, where $g_{N}=\log_{2}N$ is the chain-length exponent and $g_{N_{0}}$ is the one for the target chain. Choosing an appropriate $w_{1}$ yields a good collapse. In this study, we took N=512 as the target chain, so $g_{N_{0}}=9$. Panels (a1)–(a3) and (b1)–(b3) of Figure 5 show the resulting curve collapses for the 3D and 2D cases, respectively, under various $\phi_{d}$ conditions. The time axis is defined as $t_{w_{1}}=t\times w_{1}^{g_{N_{0}}-g_{N}}$.

The optimal scaling factors that yield the best data collapse are $w_{1}=1.81$, 1.97, and 2.19 for $\phi_{3}=0.4$, 0.2, and 0.1 in the 3D study, and $w_{1}=2.31$, 3.04, and 3.44 for $\phi_{2}=0.6$, 0.3, and 0.15 in the 2D study. To determine the optimal $w_{1}$, we calculated the mean distribution width $\langle W_{1}\rangle$ during the first expansion stage over a range of trial $w_{1}$ values and selected the value that minimized the width. Panels (a4) and (b4) present the variation in $\langle W_{1}\rangle$ vs. $w_{1}$ for different $\phi_{d}$ in the two dimensionalities.

**Figure 6:**
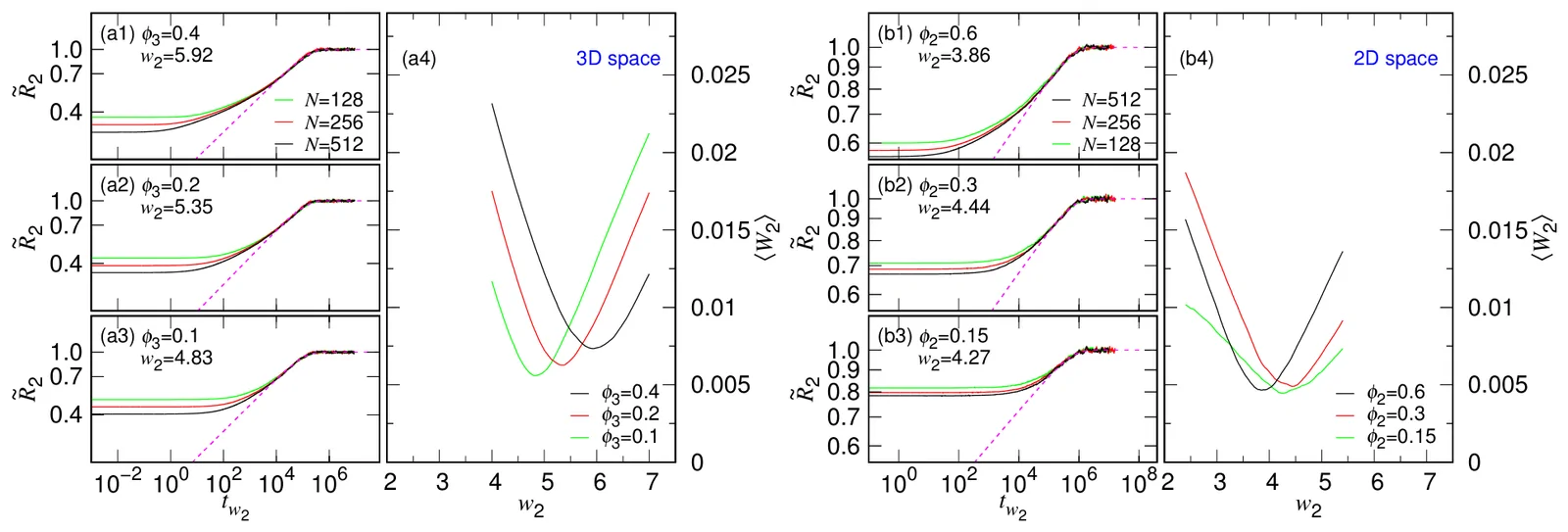
Collapse of the ${\tilde{R}}_{2}$ vs. $t_{w_{2}}$ curves in the second stage for (**a1**–**a3**) the 3D expansion cases and (**b1**–**b3**) the 2D expansion cases. The confining volume fraction $\phi_{d}$ and the $w_{2}$ value used to achieve the collapse are indicated in each panel. The chain length *N* is shown in the legends. The magenta dashed line represents a fit obtained via the equation ${\tilde{R}}_{2}\left(t\right)={\left(1-\exp\left(-\frac{t}{\tau_{2}}\right)\right)}^{\alpha_{2}}$. The variations in $\langle W_{2}\rangle$ vs. $w_{2}$ in the two spatial dimensions for the different $\phi_{d}$ are plotted in Panels (**a4**,**b4**).

Similarly, the collapse of the ${\tilde{R}}_{2}$ curves, defined by $R/R_{F}$, in the second stage is achieved by introducing a second time variable $t_{w_{2}}=t\times w_{2}^{g_{N_{0}}-g_{N}}$ and choosing $w_{2}$ such that the width of the collapsed distribution, $\langle W_{2}\rangle$, is minimized. The results are shown in Figure 6.

For the 3D case, the optimal values are $w_{2}=5.92$, 5.35, and 4.83 for $\phi_{3}=0.4$, 0.2, and 0.1, respectively. With these choices, the sigmoidal portions around the upper bending region collapse into a tight band (see Panels (a1)–(a3)). In 2D, the optimal scaling factors are $w_{2}=3.86$, 4.44, and 4.27 for the corresponding $\phi_{2}$ values, as illustrated in Panels (b1)–(b3). The variations in the mean width $\langle W_{2}\rangle$ as a function of $w_{2}$ are shown in Panels (a4) and (b4), and the results were used to determine the optimal $w_{2}$.

To quantify the evolution of the chain size, the collapsed data were fitted to the following two analytical forms: ${\tilde{R}}_{1}\left(t\right)={\left(1+\frac{t}{\tau_{1}}\right)}^{\alpha_{1}}$ for the first expansion stage, and ${\tilde{R}}_{2}\left(t\right)={\left(1-\exp\left(-\frac{t}{\tau_{2}}\right)\right)}^{\alpha_{2}}$ for the second expansion stage. The obtained fit curves have been plotted as magenta dashed lines overlaying the collapsed data bands in Figure 5 and Figure 6, Panels (a1)–(a3) and (b1)–(b3). The fits capture the variations in both stages with high precision.

**Figure 7:**
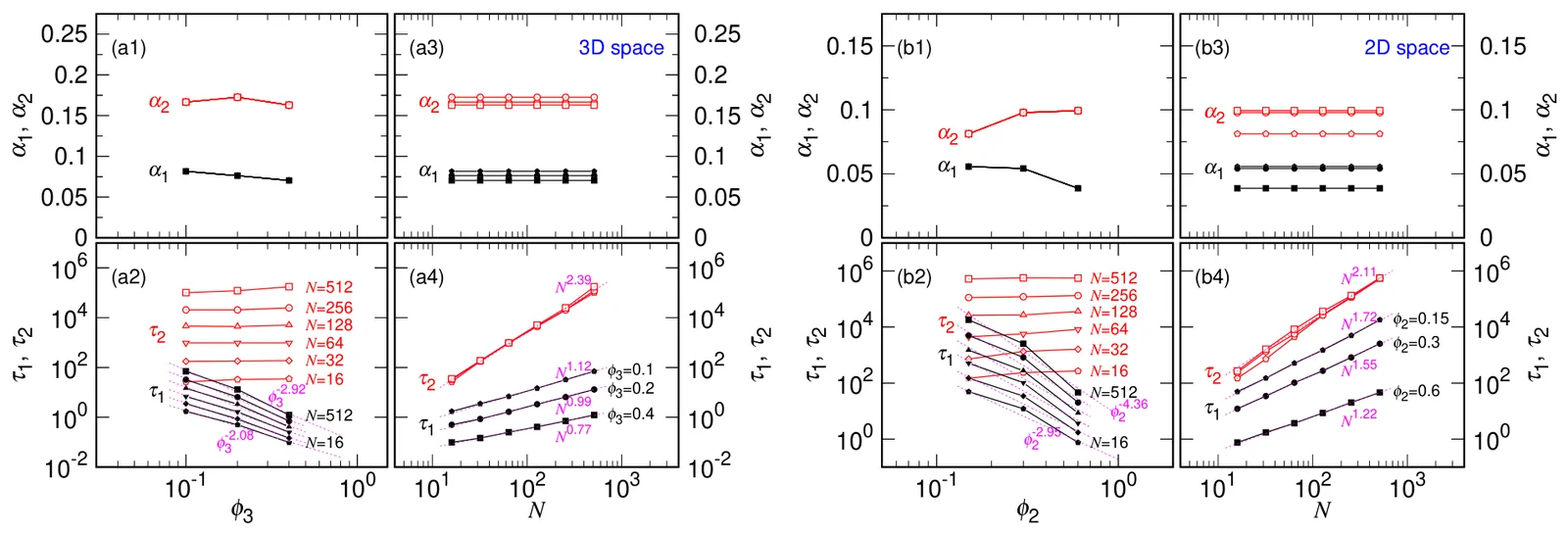
Fitting parameters $\alpha_{1}$, $\alpha_{2}$, $\tau_{1}$, and $\tau_{2}$ plotted against $\phi_{d}$ for a given chain length *N* in *d*-dimensional space are shown in panels (**a1**,**a2**) for d=3 and (**b1**,**b2**) for d=2. The same parameters plotted against *N* for a fixed $\phi_{d}$ are presented in panels (**a3**,**a4**,**b3**,**b4**).

The obtained fit parameters $\alpha_{1}$ and $\alpha_{2}$ versus $\phi_{3}$ and $\phi_{2}$ are plotted in Figure 7, Panels (a1) and (b1).

We observe that the exponent $\alpha_{1}$ slightly decreases as $\phi_{3}$ or $\phi_{2}$ increases, whereas $\alpha_{2}$ remains essentially constant for the 3D case and grows with $\phi_{2}$ in the 2D expansion. To probe the characteristic times $\tau_{1}$ and $\tau_{2}$, we performed a one-parameter fit of the two analytic forms for chains of various lengths, keeping the previously obtained values of $\alpha_{1}$ and $\alpha_{2}$ fixed. The resulting $\tau_{1}$ and $\tau_{2}$ are displayed in Panels (a2) and (b2). As expected, a longer chain exhibits a larger characteristic time. For a given chain length *N*, $\tau_{2}$ is roughly constant, whereas $\tau_{1}$ decreases when $\phi_{3}$ or $\phi_{2}$ increase, reflecting the effect of stronger confinement at beginning. A least-squares fit of the relation $\tau_{1}∼\phi_{d}^{\;p_{1}}$ gives the exponent $p_{1}$. In the 3D study, $p_{1}$ is −2.08 for the short chain (N=16) and decreases to −2.92 for the long chain (N=512). In the 2D case, the exponent is −2.95 and reduces to −4.36 for the same variation in chain length.

Because $\alpha_{1}$ and $\alpha_{2}$ were extracted from the collapsed curves in Figure 5 and Figure 6, they appear as fixed values plotted against *N* in Panels (a3) and (b3). The characteristic times, on the other hand, grow with *N* for a given $\phi_{d}$, as shown in Panels (a4) and (b4). The dependence follows a power law: $\tau_{1}∼N^{s_{1}}$ and $\tau_{2}∼N^{s_{2}}$. The fitted exponents are $s_{1}=0.77$, 0.99, 1.12 for $\phi_{3}=0.4$, 0.2, 0.1, and $s_{1}=1.22$, 1.55, 1.72 for $\phi_{2}=0.6$, 0.3, 0.15. These exponents are directly related to the time-shifting factor $w_{1}$ via $w_{1}=2^{s_{1}}$. The corresponding values derived from the $s_{1}$ measurements are $w_{1}=1.71$, 1.98, 2.30 for the 3D space and $w_{1}=2.35$, 3.14, 3.43 for the 2D space, which agree well with the $w_{1}$ values obtained from the curve-collapse analysis. For the second expansion stage, we find $s_{2}=2.39$ for the 3D expansion and $s_{2}=2.11$ for the 2D one, corresponding to $w_{2}=5.24$ and $w_{2}=4.32$, respectively. These are again close to the mean $w_{2}$ values of 5.37 and 4.19 obtained in Figure 6. The consistency between the time-shifting factors and the fitting values confirms the coherence of our analysis.

A direct comparison between the two dimensionalities reveals that the exponents $\alpha_{1}$ and $\alpha_{2}$ are larger for 3D expansion than for 2D, whereas the characteristic times $\tau_{1}$ and $\tau_{2}$ are smaller in 3D. Consequently, the evolution shows that 3D expansion proceeds faster than 2D. The main reason is that in 2D, the expansion is confined to a plane, which restricts the motion and therefore slows down the dynamics.

### 3.3. Expansion Speed at $T_{\theta }$

**Figure 8:**
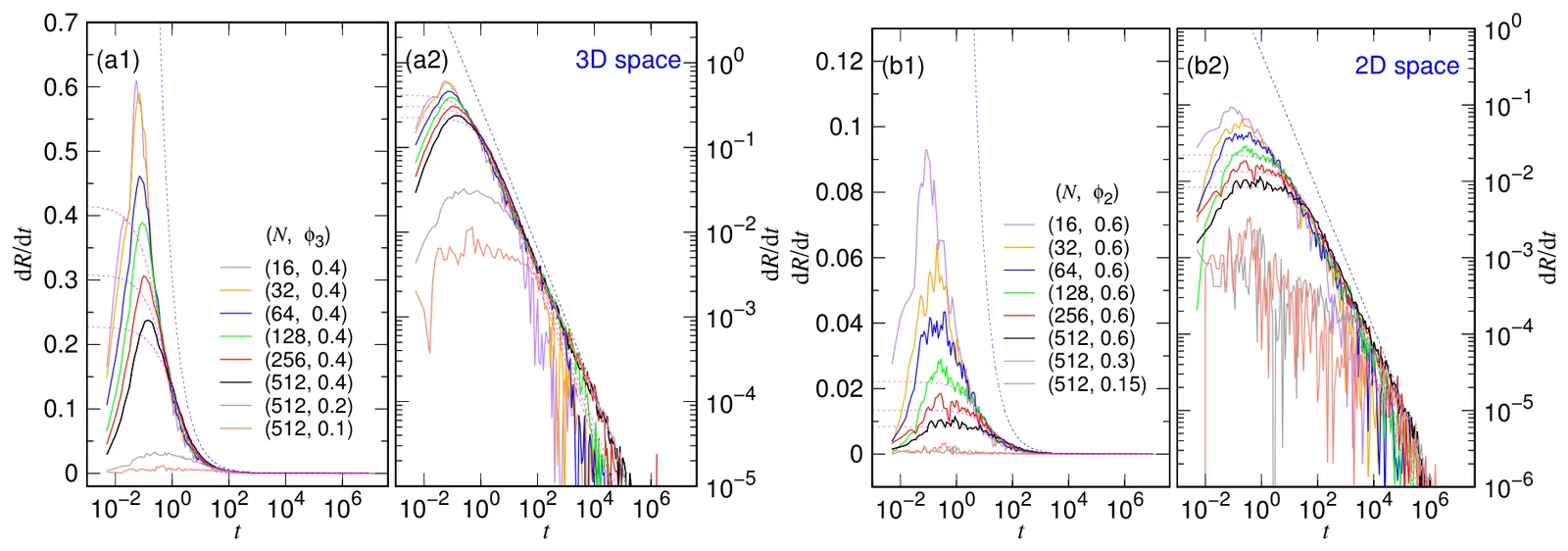
Expansion speed dR/dt versus time in Panels (**a1**,**a2**) for the 3D case, and in Panels (**b1**,**b2**) for the 2D case. The chain length *N* and the initial volume fraction $\phi_{d}$ are indicated in the legends. The magenta dashed line represents the predicted first-stage speed $V_{1}\left(t\right)$, while the blue dashed line represents the predicted second-stage speed $V_{2}\left(t\right)$ described in the main text. Panels (**a2**,**b2**) show the log–log versions of Panels (**a1**,**b1**), respectively.

The expansion speed indicates how quickly a chain returns to its natural size after the confining wall is removed. It can be studied by numerically computing the time derivative of R(t) given in Figure 4. The results are presented in Figure 8.

Panels (a1) and (b1) show that the speed curves exhibit a pronounced peak during the initial stage, occurring at t<10 for the 3D expansion and at t<100 for the 2D expansion. The appearance of the peak reflects that the chain is initially confined within a cavity and therefore has zero speed. Once the constraint is removed, the strong internal pressure is released, driving the chain into an explosive expansion. We found that under the same confining conditions, the peak speed decreases as the chain length increases, as illustrated by the curves at $\phi_{3}=0.4$ and $\phi_{2}=0.6$. Conversely, for a fixed chain length (e.g., N=512 in the plots), decreasing the confinement parameter $\phi_{d}$ reduces the height of the peak.

For comparison, the predicted first-stage speed $V_{1}\left(t\right)=\alpha_{1}\frac{R_{I}}{\tau_{1}}{\left(1+\frac{t}{\tau_{1}}\right)}^{\alpha_{1}-1}$ is plotted as magenta dashed lines for N=128, 256, and 512, using the fitting parameters extracted from Figure 7. This equation was derived for the course of the expansion; therefore it does not capture the transition dynamics that occur at the instant the confining constraint is removed. Nevertheless, it predicts an initial speed $\alpha_{1}\frac{R_{I}}{\tau_{1}}$ that is quite close to the observed peak speed. We know that under strong confinement, the initial radius scales as $R_{I}∼N^{1/d}$ while the characteristic time scales as $\tau_{1}∼N^{s_{1}}$ (see Panels (a4) and (b4) of Figure 7). Because the exponent $s_{1}$ exceeds 1/d, the peak height decreases as *N* increases at a given confinement $\phi_{d}$, which is exactly what has been observed in Figure 8. If the chain length is held fixed, Panels (a2) and (b2) of Figure 7 have shown that $\tau_{1}$ decreases as $\phi_{d}$ is increased. It reflects the fact that a more loosely confined globule expands less violently than a tightly confined one, thereby yielding a longer characteristic time for the first-stage expansion. Using the scaling relation $R_{I}∼\phi_{d}^{-1/d}$, a simple calculation shows that the peak speed should decrease as the confinement strength $\phi_{d}$ is reduced.

The predicted second-stage speed $V_{2}\left(t\right)=\alpha_{2}\frac{R_{F}}{\tau_{2}}{\left(1-\exp\left(-\frac{t}{\tau_{2}}\right)\right)}^{\alpha_{2}-1}\exp\left(-\frac{t}{\tau_{2}}\right)$ is plotted as a blue dashed line for N=512. It well captures the speed profile in the long-time regime. To highlight the variation in the low-speed region, the figures are replotted on a log–log scale (Panels (a2) and (b2)). It is worth noticing that the speed curves for different *N* share a common decreasing trend, closely following the blue dashed lines.

A comparison across dimensionalities, regardless of the different confining conditions $\phi_{d}$, shows that the expansion speed in 3D is about an order of magnitude faster than in 2D. It confirms again that the dynamics in 2D are slower, owing to the restriction in the available space for motion.

**Figure 9:**
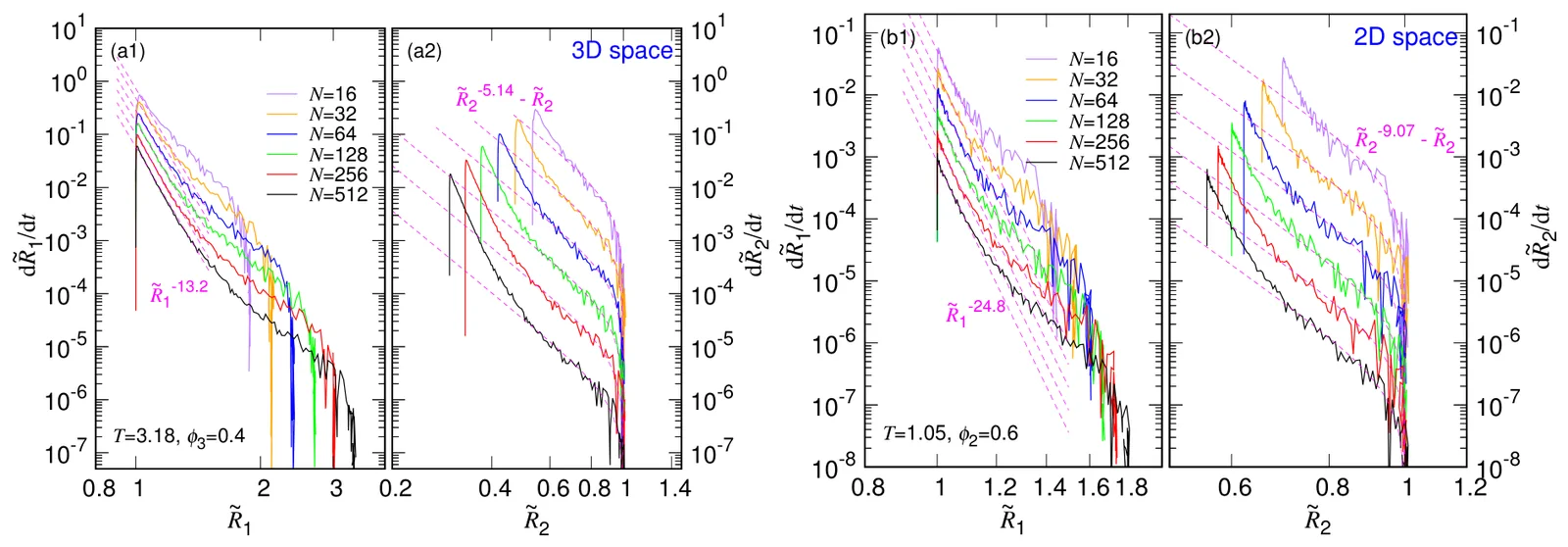
$d{\tilde{R}}_{1}/dt$ versus ${\tilde{R}}_{1}$ is plotted in Panels for (**a1**) the 3D and (**b1**) the 2D expansion cases, while $d{\tilde{R}}_{2}/dt$ versus ${\tilde{R}}_{2}$ is shown in Panels (**a2**,**b2**). The confinement conditions are $\phi_{3}=0.4$ for the 3D case and $\phi_{2}=0.6$ for the 2D case. The chain length *N* is indicated in the legends.

We then investigate the kinetic behavior of the system by examining how the expansion speed of chain varies with the chain size. It describes the change of the chain state during an expansion process. The results, $d{\tilde{R}}_{1}/dt$ vs. ${\tilde{R}}_{1}$ and $d{\tilde{R}}_{2}/dt$ vs. ${\tilde{R}}_{2}$, in the 3D and 2D spaces are presented in Figure 9. Here, ${\tilde{R}}_{1}=\frac{R}{R_{I}}$ and ${\tilde{R}}_{2}=\frac{R}{R_{F}}$ denote, respectively, the ratio of the chain’s expansion relative to its initial size $R_{I}$ and the degree of recovery towards the equilibrium chain size $R_{F}$ in the solution.

We observe that, for a given $\phi_{3}$ or $\phi_{2}$, the $\frac{d{\tilde{R}}_{1}}{dt}$ curves display a sharp surge that begins from the zero velocity at ${\tilde{R}}_{1}=1$. This indicates an explosive expansion occurred immediately after the release. In the small-${\tilde{R}}_{1}$ region, the curves exhibit a parallel, decreasing trend that depends on the chain length, as illustrated in Panels (a1) and (b1). This behavior suggests a power-law variation consistent with the kinetic prediction:

(7) $$\frac{d{\tilde{R}}_{1}}{dt}=\frac{\alpha_{1}}{\tau_{1}}{\tilde{R}}_{1}^{1-\frac{1}{\alpha_{1}}}$$

The magenta dashed lines, overlaid on the data, represent the results of a one-parameter fit using Equation (7) in which $\tau_{1}$ is the only free parameter and $\alpha_{1}$ is fixed at the values obtained from Figure 5: $\alpha_{1}=0.0705$ for the 3D case and $\alpha_{1}=0.0387$ for the 2D case. We observe that the $\frac{d{\tilde{R}}_{1}}{dt}$ curves follow the predicted scaling very closely, namely, ${\tilde{R}}_{1}^{-13.2}$ for the 3D and ${\tilde{R}}_{1}^{-24.8}$ for the 2D case.

Similarly, the speed $\frac{d{\tilde{R}}_{2}}{dt}$ displays a hump-shaped curve that exhibits a parallel behavior for different chain lengths in Panels (a2) and (b2), with a sharp drop to zero as ${\tilde{R}}_{2}$ approaches 1. The magenta dashed curves represent the results of a fit in which the only free parameter is $\tau_{2}$ in the following equation:

(8) $$\frac{d{\tilde{R}}_{2}}{dt}=\frac{\alpha_{2}}{\tau_{2}}[{\tilde{R}}_{2}^{1-\frac{1}{\alpha_{2}}}-{\tilde{R}}_{2}]$$

Here, $\alpha_{2}$ is fixed at the values obtained from the $\phi_{3}=0.4$ and $\phi_{2}=0.6$ cases in Figure 6, namely, $\alpha_{2}=0.163$ and $\alpha_{2}=0.0993$, respectively. The simulation data closely follow the fitted curves, which exhibit the scaling forms ${\tilde{R}}_{2}^{-5.14}-{\tilde{R}}_{2}$ in 3D and ${\tilde{R}}_{2}^{-9.07}-{\tilde{R}}_{2}$ in 2D expansion.

It is worth noting that the variation curves for different chain lengths can be collapsed onto a single master curve in each of the two expansion stages if the time variable is scaled by the corresponding characteristic time: ${\tilde{t}}_{1,2}=t/\tau_{1,2}$. This collapse shows that the dynamics follow two universal paths in the velocity vs. chain-size plot, which are expressed as

(9) $$\begin{array}{ccc} \frac{d{\tilde{R}}_{1}}{d{\tilde{t}}_{1}} & = & \alpha_{1}{\tilde{R}}_{1}^{1-\frac{1}{\alpha_{1}}}\;\mathrm{for}\;\mathrm{the}\;\mathrm{first}\;\mathrm{stage}; \end{array}$$

(10) $$\begin{array}{ccc} \frac{d{\tilde{R}}_{2}}{d{\tilde{t}}_{2}} & = & \alpha_{2}[{\tilde{R}}_{2}^{1-\frac{1}{\alpha_{2}}}-{\tilde{R}}_{2}]\;\mathrm{for}\;\mathrm{the}\;\mathrm{second}\;\mathrm{stage}. \end{array}$$

### 3.4. Free Energy and Expansion Exponents

In the preceding subsection, we demonstrated that the expansion kinetics are well described by Equations (7) and (8). These results enable us to determine the free energy via the integral $F=-\int \eta_{\mathrm{eff}}\left(\frac{dR}{dt}\right)dR$. For the first stage of expansion, the free energy is obtained as

(11) $$F_{1}∼k_{B}TN^{\frac{2+y_{1}}{d}+x_{1}-s_{1}}\frac{1}{\frac{1}{\alpha_{1}}-2-y_{1}}{\tilde{R}}_{1}^{2+y_{1}-\frac{1}{\alpha_{1}}}$$

where we have employed the scaling relation $R_{I}∼\sigma N^{1/d}$ for the initial chain size and the time scale $\tau_{1}∼\Delta t_{r}\;N^{s_{1}}$, with $\Delta t_{r}=\eta_{0}\sigma^{2}/k_{\;B}T$ representing the Rouse time. We also assumed that the effective friction coefficient varies with *N* and *R* during the expansion as $\eta_{\mathrm{eff}}∼\eta_{0}N^{x_{1}}{\left(\frac{R}{\sigma }\right)}^{y_{1}}$. Blob theory predicts that the free energy of a globular chain takes the form of

(12) $$F∼k_{B}T{\left(\frac{\sigma N^{\nu_{b}}}{R}\right)}^{dz}∼k_{B}TN{\tilde{R}}_{1}^{-dz}$$

where *R* is the globule size, $z=\frac{1}{d\nu_{b}-1}$ is the des Cloizeaux exponent in *d* dimensions [64], and $\nu_{b}$ is the Flory exponent in a blob. The consistency between the two free-energy expressions requires the following identities for the exponents:

(13) $$\begin{array}{ccc} \frac{2+y_{1}}{d}+x_{1}-s_{1} & = & 1 \end{array}$$

(14) $$\begin{array}{ccc} \frac{1}{\alpha_{1}}-2-y_{1} & = & dz \end{array}$$

Within the picture of a radial expansion, one can further argue that $y_{1}=-1$ [35]. Using this value together with the numerically obtained values of $s_{1}$ and $\alpha_{1}$, the remaining exponents $x_{1}$ and $\nu_{b}$ can be calculated. The results are given in Table 1.

Similarly, in the second stage of expansion, the free energy can be obtained from $F_{2}=-\int \eta_{\mathrm{eff}}R_{F}^{2}\frac{d{\tilde{R}}_{2}}{dt}d{\tilde{R}}_{2}$, which yields

(15) $$F_{2}∼k_{B}TN^{x_{2}+y_{2}\nu_{\theta }+2\nu_{\theta }-s_{2}}\left[\frac{1}{2+y_{2}}{\tilde{R}}_{2}^{2+y_{2}}+\frac{1}{\frac{1}{\alpha_{2}}-2-y_{2}}{\tilde{R}}_{2}^{2+y_{2}-\frac{1}{\alpha_{2}}}\right].$$

Here, we have used $R_{F}∼\sigma N^{\nu_{\theta }}$ for the theta condition in *d*-dimensional space and $\tau_{2}∼\Delta t_{r}\;N^{s_{2}}$, and assumed $\eta_{\mathrm{eff}}∼\eta_{0}\;N^{x_{2}}\;{\left(\frac{R}{\sigma }\right)}^{y_{2}}$. The first term on the right-hand side of $F_{2}$ corresponds to the well-known entropic free energy of a single chain, $k_{B}T\frac{R^{2}}{N\sigma^{2}}$, or equivalently $k_{B}T\;N^{2\nu_{\theta }-1}{\tilde{R}}_{2}^{2}$. Comparing the two expressions gives

(16) $$\begin{array}{ccc} y_{2} & = & 0 \end{array}$$

(17) $$\begin{array}{ccc} x_{2}-s_{2} & = & -1. \end{array}$$

The exponent $x_{2}$ can be then calculated and the result is given in Table 1.

The errors of $s_{1}$ and $s_{2}$ reported in the table are estimated from the linear least-squares fit to the log–log plots in Figure 7(a4,b4). The errors for $\alpha_{1}$ and $\alpha_{2}$ are obtained by nonlinear least-squares curve fitting in Figure 5 and Figure 6 using the Levenberg–Marquardt algorithm. The confidence level for the error estimates is 95%. The errors for $x_{1}$, $x_{2}$, and $\nu_{b}$ are calculated by error propagation, using Equations (13), (14) and (17).

As shown, the exponent $x_{1}$ exceeds unity in both spatial dimensions, and a similar trend is observed for $x_{2}$. This indicates that the short-range attractive interactions between beads in a θ-solution play a crucial role in determining the effective friction coefficient associated with the expansion speed. In the earlier study that used a freely jointed chain of phantom beads [35], both $x_{1}$ and $x_{2}$ were found to be equal to one.

This analysis allows us to evaluate the effective Flory exponent $\nu_{b}$ inside a blob during the first stage of expansion. The resulting values are approximately 0.41 for the 3D expansions and 0.54 for the 2D ones, reflecting the degree of confinement of the chain within the cavity at the onset of expansion.

Within the ideal-chain framework, the exponents $\alpha_{1}$ and $\alpha_{2}$ are predicted to be $\frac{1}{3}$ and $\frac{1}{4}$, respectively, regardless of spatial dimension. Table 1 reveals that the measured values for these exponents differ markedly from the ideal predictions and exhibit a strong dependence on the dimensionality *d*. Consequently, the realistic kinetics of chain expansion under theta conditions cannot be captured by the simple ideal, freely jointed chain model.

It is worth noting that in the studies based on the freely jointed chain model, the free energy of an ideal chain has been shown to take the form $F∼k_{B}T\left[\frac{R^{2}}{N\sigma^{2}}+\frac{N\sigma^{2}}{R^{2}}\right]$ in the second stage when the chain restores to its natural state [35,65]. In contrast, the bead--spring chain model studied here suggests that the free energy should be $F∼k_{B}TN^{2\nu_{\theta }-1}[{\tilde{R}}_{2}^{2}+{\tilde{R}}_{2}^{2-\frac{1}{\alpha 2}}]$, or equivalently, $F∼k_{B}T[\frac{R^{2}}{N\sigma^{2}}+{\left(\frac{\sigma }{R}\right)}^{\frac{1}{\alpha 2}-2}N^{\frac{\nu_{\theta }}{\alpha_{2}}-1}]$. Minimizing this free energy through ${\left.\frac{dF}{dR}\right|}_{R_{\mathrm{eq}}}=0$ indeed yields the expected scaling relation at the θ-point, $R_{\mathrm{eq}}∼\sigma N^{\nu_{\theta }}$. For the 3D expansion released from the high compression condition $\phi_{3}=0.4$, the exponents appearing in the second term of the free energy are $\frac{1}{\alpha 2}-2=4.14\;\left(6\right)$ and $\frac{\nu_{\theta }}{\alpha_{2}}-1=2.07\;\left(3\right)$ as obtained from the simulations. For the 2D expansion with $\phi_{2}=0.6$, the corresponding exponents are 8.07(35) and 4.75(19), respectively. These results indicate that the free-energy landscape of the bead--spring chain is markedly different from that of a freely jointed chain. The discrepancy originates evidently from the fact that the chain segments cannot overlap or penetrate each other. Consequently, although the effective interactions between monomers cancel out under theta conditions, the free-energy-related properties cannot be inferred directly from an idealized phantom-chain model. How the non-overlapping and non-penetrating constraints influence the free energy deserves a detailed study in the future.

## 4. Conclusions

Molecular simulations with a Langevin thermostat were performed to investigate the expansion kinetics of single chains released from a spherically confined cavity under theta conditions. The chains were modeled as bead--spring chains, which preserve local attractive interactions and prevent segment crossing. First, we determined the θ-temperature by studying the scaling of the chain size and searching for the expected power-law behavior $R∼\sigma N^{\nu_{\theta }}$ with $\nu_{\theta }=\frac{1}{2}$ in 3D and $\nu_{\theta }=\frac{4}{7}$ in 2D. The resulting θ-temperatures were $T_{\theta }=3.18$ and 1.05, respectively, for the two spatial dimensions. The study revealed how asphericity and the shape factor vary with chain length as the temperature changes. At the θ-temperature, the chain was found to adopt a coil conformation with local monomer aggregation.

The chain expansion was then investigated at the θ-temperature by systematically varying the chain length *N* and the confining volume fraction $\phi_{d}$. To suppress thermal fluctuations, 1000 independent runs were performed for each $\left(N,\phi_{d}\right)$ pair, allowing for robust statistical analysis. The averaged evolution of the chain size showed a sigmoidal variation in a log--log plot, suggesting a two-stage transition that evolves from the initially confined state with size $R_{I}$ to the final state with size $R_{F}$. These evolution curves exhibited remarkable regularity across different chain lengths, and a suitable rescaling of the time axis collapsed the curves onto master curves. For example, in the study of ${\tilde{R}}_{1}\equiv R/R_{I}$ vs. $t_{\omega_{1}}$ and ${\tilde{R}}_{2}\equiv R/R_{F}$ vs. $t_{\omega_{2}}$ (see Figure 5 and Figure 6), the data collapsed onto a single master curve in each stage, described by its own characteristic time. We further demonstrated that the master curves can be well fitted by a power-law function ${\tilde{R}}_{1}\left(t\right)={\left(1+\frac{t}{\tau_{1}}\right)}^{\alpha_{1}}$ for the first stage, and by an exponential recovery function ${\tilde{R}}_{2}\left(t\right)={\left(1-\exp\;\left(-\frac{t}{\tau_{2}}\right)\right)}^{\alpha_{2}}$ for the second stage. The fitting parameters $\alpha_{1},\alpha_{2},\tau_{1},\tau_{2}$ are shown in Figure 7 for the two dimensionalities. A comparison across dimensionalities reveals that the systems exhibit faster kinetics in 3D. The exponents $\alpha_{1}$ and $\alpha_{2}$ are larger, while the characteristic times $\tau_{1}$ and $\tau_{2}$ are smaller in 3D. It can be attributed to the reduced available space for motion in 2D, where the chain movement is confined to a plane.

The expansion rate was found to increase with higher $\phi_{d}$ and smaller *N*. Plots of $\frac{d{\tilde{R}}_{1}}{dt}$ vs. ${\tilde{R}}_{1}$ and $\frac{d{\tilde{R}}_{2}}{dt}$ vs. ${\tilde{R}}_{2}$ (Figure 9) directly validate the kinetic equations for expansion given in Equations (7) and (8). Thus, universal evolutionary trajectories were identified in the “velocity vs. chain-size” phase space for both stages of expansion. Free-energy profiles can be obtained by integrating the kinetic equations over the corresponding stages. In collaboration with the blob theory and Flory’s entropic free energy, several relationships between scaling exponents were derived. The resulting exponents were summarized in Table 1. Compared with the exponents reported in the earlier study that employed an idealized FJC model at theta conditions [35], significant differences were observed. The FJC model predicts $\alpha_{1}=\frac{1}{3}$, $\alpha_{2}=\frac{1}{4}$, $s_{1}=\frac{3}{d}$, and $s_{2}=2$ for spatial dimensions d=3 and 2. The discrepancy arises from the present model’s inclusion of non-penetrating monomers and non-crossing segments, which introduce local interactions that critically influence the chain dynamics. Consequently, the expansion kinetics can be correctly described only when these effects are incorporated. The FJC model provides only an essential baseline for understanding ideal expansion at theta conditions through a phantom chain. Noticeably, the integrated free-energy functional for the BSC model in the θ solvent is found to be $F∼k_{B}T\left[\frac{R^{2}}{N\sigma^{2}}+{\left(\frac{\sigma }{R}\right)}^{\frac{1}{\alpha 2}-2}N^{\frac{\nu_{\theta }}{\alpha_{2}}-1}\right]$, in contrast with the expression for FJC: $F∼k_{B}T\left[\frac{R^{2}}{N\sigma^{2}}+\frac{N\sigma^{2}}{R^{2}}\right]$.

We remark that the equations ${\tilde{R}}_{1}\left(t\right)$ and ${\tilde{R}}_{2}\left(t\right)$ were used as empirical fitting functions in the current study. They characterize the chain-size evolution and allow extraction of the corresponding scaling exponents and characteristic times, which in turn facilitate further studies such as expansion speed, trajectory analysis in phase space, and free-energy estimation. This procedure provides a useful workflow for experimental studies: first, the chain size is measured; second, it is fitted to empirical laws; finally, the universal trajectory and underlying free-energy landscape are inferred.

In the future, we will extend this study to include hydrodynamic interactions or chain rigidity in order to further bridge the gap between simulations and real-world polymer systems, thereby paving the way for predictive modeling of complex polymeric materials as they transition from a confined to an unconfined state. Several important open questions remain worth investigating: To what extent do hydrodynamic interactions alter the expansion stages, the scaling exponents, and the characteristic times? The persistence length plays a crucial role, especially when the cavity diameter becomes comparable to it. How do the kinetics of expansion change with varying persistence length? In this study, we varied solvent quality by changing temperature. Would a study in which solvent quality changes due to modified molecular interactions produce similar master curves and universal trajectories in the “velocity vs. chain-size” phase space? What happens to the kinetics if the internal structure of the chain is altered, for example, by using hetero-monomers or by adding side-chains? How does the free-energy functional change when the model is modified in the above ways? The theta point could be investigated by other methods, such as computing the second virial coefficient. A combined analysis of the renormalization-group method and lattice self-avoiding walk models might elucidate the physical mechanism underlying the dimension-induced changes in the scaling exponents at the theta point.

## Figures and Tables

**Table 1:** Expansion exponents $s_{1}$, $\alpha_{1}$, $s_{2}$, and $\alpha_{2}$ obtained from Figure 7 in the two spatial dimensions, along with the calculated exponents $x_{1}$, $\nu_{b}$, and $x_{2}$.

dim	$\phi_{3}$	$s_{1}$	$\alpha_{1}$	$s_{2}$	$\alpha_{2}$	$x_{1}$	$\nu_{b}$	$x_{2}$
3	0.4	0.77(4)	0.070(1)	2.55(18)	0.16(1)	1.43(4)	0.41(1)	1.55(18)
3	0.2	0.99(3)	0.076(1)	2.38(9)	0.17(2)	1.65(3)	0.42(1)	1.38(10)
3	0.1	1.12(5)	0.081(5)	2.23(9)	0.17(1)	1.78(5)	0.42(1)	1.23(9)
**dim**	$\phi_{2}$	$s_{1}$	$\alpha_{1}$	$s_{2}$	$\alpha_{2}$	$x_{1}$	$\nu_{b}$	$x_{2}$
2	0.6	1.22(3)	0.039(1)	1.98(16)	0.099(3)	1.72(3)	0.54(1)	0.98(16)
2	0.3	1.55(8)	0.054(2)	2.20(7)	0.098(4)	2.05(9)	0.56(1)	1.20(7)
2	0.15	1.72(9)	0.056(2)	2.16(9)	0.081(3)	2.22(9)	0.56(1)	1.16(9)

## Data Availability

The original contributions presented in this study are included in the article. Further inquiries can be directed to the corresponding author.
